# Folate-Targeted Liposomal Formulations Improve Effects of Methotrexate in Murine Collagen-Induced Arthritis

**DOI:** 10.3390/biomedicines10020229

**Published:** 2022-01-21

**Authors:** Diana Guimarães, Franck Lager, Gilles Renault, Jamil Guezguez, Michael Burnet, Joana Cunha, Artur Cavaco-Paulo, Eugénia Nogueira

**Affiliations:** 1 CEB—Centre of Biological Engineering, University of Minho, 4710-057 Braga, Portugal; dianapguimaraes@ceb.uminho.pt (D.G.); joanacunha@ceb.uminho.pt (J.C.); 2 LABBELS—Associate Laboratory, University of Minho, 4710-055 Braga, Portugal; 3 SOLFARCOS—Pharmaceutical and Cosmetic Solutions, 4710-053 Braga, Portugal; 4 INSERM—Institut National de la Santé et de la Recherche Médicale, U1016, Institut Cochin, 75014 Paris, France; franck.lager@inserm.fr (F.L.); gilles.renault@inserm.fr (G.R.); 5 Synovo GmbH, Paul Ehrlich Str. 15, D-72076 Tübingen, Germany; jamil.guezguez@synovo.com (J.G.); michael.burnet@synovo.com (M.B.)

**Keywords:** liposomes, methotrexate, folate-targeting, rheumatoid arthritis, collagen-induced arthritis

## Abstract

Methotrexate (MTX) is first-line therapy for the treatment of rheumatoid arthritis (RA), however, its use may be limited by side effects notably post-injection malaise. When patients are intolerant or become unresponsive, second-line or antibody therapy may be indicated. A folate-targeted liposomal formulation of MTX (FL-MTX) is tropic to arthritic paws and prevents the onset of collagen-induced arthritis (CIA) in the mouse. We optimized the drug-to-lipid molar ratio to 0.15 and demonstrated the therapeutic efficacy of this form at 2 mg/kg MTX intraperitoneal (i.p.) twice a week. These improved liposomes were present in inflamed joints in proportion to the degree of swelling of the paw and bone remodeling activity. FL-MTX had lower hepatic and renal elimination of MTX than the free substance. FL-MTX provided equivalent results when given i.p. or subcutaneous (s.c.) and FL-MTX 2 mg/kg (drug/lipid 0.15), twice weekly, was similar to or more effective than 35 mg/kg MTX (same route and schedule) in reducing the incidence and swelling in the murine CIA model. These results suggest that FL-MTX is a more potent nanotherapeutic formulation than free MTX treatment. Its potential benefits for patients may include reduced frequency of treatment and lower overall doses for a given response.

## 1. Introduction

Rheumatoid arthritis (RA) is considered the commonest type of chronic inflammatory arthritis, characterized by the inflammation of the joints. This process leads to synovial hyperplasia by the infiltration of activated immune cells promoting cartilage and bone destruction [1]. RA is a common cause of disability. Mortality rates in RA patients (1.28–2.98%) are higher than in the general population [2]. Patients that develop adverse effects with typical drugs used in RA treatment have their life expectancy reduced in 3–5 years [2,3]. Patients with RA have a higher risk of acute cardiovascular events, compared with the general population [4]. Methotrexate (MTX) is the anchor drug in first-line therapy indicated for the treatment of RA [5,6,7]. However, careful monitoring of the patients is required to adjust the dose and respond to treatment-related effects. Minor toxic effects, such as stomatitis, malaise, nausea, diarrhea, headaches, and mild alopecia, are common but improve with folic acid (FA, folate) supplementation [6,8]. Other more serious effects include gastrointestinal or bone marrow toxicity, pneumonitis, hepatotoxicity, and cirrhosis [7]. Clinical reports refer that 10–30% of the patients taking MTX need to discontinue within 1–2 years of initiation [9,10,11]. If patients show moderate or high disease activity after 3–6 months of therapy despite dose optimization, another DMARD (disease-modifying anti-rheumatic drug, e.g., leflunomide, sulfasalazine, hydroxychloroquine, azathioprine) or a biologic agent (anti-TNF antibodies) added or substituted into the therapeutic scheme [12]. Despite their clinical effectiveness, the use of biological agents is limited by cost and public payers tend to prefer DMARDs for budgetary reasons [1,13]. RA therapies, while intended to reduce joint inflammation, act systemically leading to undesirable events that increase the risk of side effects. Then, it is a requirement for the improvement of the disease control measurements, as well as strategies to enhance the target therapies to act selectively on the tissues affected [14]. Activated macrophages constitute the key effector cells in RA, being reported an association between the level of macrophage activity and the intensity of joint inflammation, articular pain, and bone erosion [15]. Activated macrophages express an isoform of the receptor for the vitamin folic acid, the folate receptor (FR)β. Since few cell types express this receptor, FRβ-macrophages accumulating in arthritic joints can be targeted using folate-linked imaging and therapeutic agents [16]. Hence, folate-targeted therapies are selectively recognized by the pathogenic cell type at the sites of inflammation to the detriment of non-activated macrophages. Moreover, being activated macrophages the only population of white blood cells appears to express a functional FRβ, the cellular toxicity of these therapies is very low [17]. Liposomes represent a powerful carrier for a broad spectrum of drugs. Being in your composition substances presents in biological membranes, liposomes are both non-toxic and biodegradable [18]. Bioactive compounds encapsulated within liposomes are partially protected from dilution or degradation, representing then an excellent delivery system for their transport to pathogenic cells [19,20]. We previously reported the encapsulation of MTX in a new liposomal formulation (FL-MTX) containing a hydrophobic fragment of the surfactant protein D conjugated to a spacer and folic acid, which increase the tolerance and efficacy of the drug [21]. Our delivery system showed higher efficiency than the traditional system where the link of FA to liposomes is done by polyethylene glycol (PEG) [21]. We tested the specificity of these new liposomes in murine collagen-induced arthritic (CIA) mice, a model of RA [22]. These liposomes were present in arthritic joints to a greater degree than neighboring tissue. Analysis of the cell populations retrieved from these joints showed that macrophages with a higher FRβ expression have a greater uptake of folate-targeted to the detriment of non-targeted liposomes. Administration of FL-MTX as a prophylactic treatment, i.e., before disease signs but after induction of disease, prevented the onset of clinical signs of arthritis. In comparison, the free soluble form of MTX showed only a marginal effect when injected into the prophylactic scheme [23]. In support of first-in-human (FiH) clinical trials with FL-MTX, we optimized the drug-to-lipid ratio, the route of injection, and the dose needed to prevent disease onset in the CIA model. Finally, the biodistribution pattern of labeled liposomes was evaluated using imaging approaches to measure its accumulation in arthritic paws. These data show that MTX in a liposomal form FL-MTX exerts enhanced effects on the intended target, the arthritic paws.

## 2. Materials and Methods

### 2.1. Materials

1,2-dioleoyl-sn-glycero-3-phosphoethanolamine (DOPE) and 1,2-distearoyl-sn-glycero-3-phosphoethanolamine-n-[methoxy(polyethylene glycol)-2000] (DSPE–mPEG) were obtained from Lipoid GmbH (Ludwigshafen, Germany) and diethylene-triamine-pentaacetate (DTPA) was obtained from Avanti (Tonawanda, NY, USA). Folate-peptide was synthesized by CSBio (Menlo Park, CA, USA). All the other chemicals involved in this work were purchased from Sigma-Aldrich (Burlington, MA, USA), except MTX which was acquired from Huzhou Zhanwang Pharmaceutical (Huzhou City, China), cholesterol from Anhui Chem-Bright Bioengineering Co., Ltd. (Huaibei, China), bovine collagen type II (CII) purchased from Chondrex, Morwell Diagnostics (Zurich, Switzerland) and complete Freund’s adjuvant used in in vivo experiments from Fisher Scientific (Illkirch-Graffenstaden, France). All compounds were used without further purification.

### 2.2. Liposomes Preparation

Liposomes composed of DOPE/Cholesterol/DSPE-mPEG (54:36:10, molar ratio) [21] were produced by a pre-concentration ethanol injection method [24]. Briefly, the lipid components were dissolved in ethanol (20% of the final volume) to obtain a 1:1 initial ratio of organic:aqueous phase (*v*/*v*). The organic phase was added to an aqueous phase containing the drug MTX and folate-peptide (0.75% *w*/*v*) dissolved in phosphate-buffered saline (PBS) buffer, under vigorous magnetic stirring, at 70 °C. After ethanol reduction, the liposomal suspension was diluted five times with PBS buffer. The non-encapsulated MTX and residual ethanol were removed from the liposomes after passage through a gel filtration chromatography column (GE Healthcare, Hatfield, UK), with 5 kDa cut-off (PD-10 Desalting Columns containing 8.3 mL of Sephadex™ G-25 Medium). After separation, the concentration of the encapsulated MTX was determined by measuring the absorbance at 303 nm, the maximum wavelength of MTX in PBS. The data were recorded on spectrophotometer BioTek Synergy™ HT (Winooski, VT, USA) using a quartz microplate.

### 2.3. Determination of Size Distribution

The determination of liposome size distribution was performed using the dynamic light scattering technique. The analysis was conducted at pH 7.4 (PBS buffer) and 25.0 °C, using a Malvern Zetasizer Nano ZS (Malvern Instruments, Worcestershire, UK) by photon correlation spectroscopy. The viscosity and refractive index of the dispersant were 0.8616 cP and 1.332, respectively. Each sample was measured in triplicate and results are presented as mean value ± standard deviation (SD).

### 2.4. In Vivo Studies

All animal experiments were conducted following the European and French regulations and were approved by the local Ethics Committee (CEEA 34, Paris Descartes University) and registered by the French ministry of research under reference #9696.

Male 6-week-old DBA/1 mice, which are susceptible to CIA, were purchased from Janvier (Le Genest-St-Isle, France). To point out, the incidence in males is stronger, from our experience as well as from various publications using this mouse model [25]. Upon reception, animals were divided into groups of 6 individuals and placed in individually ventilated disposable cages (IVC Mouse Rack system, Innovive France, Le Havre, France). The cages (Disposable Cages 101) were provided with standard litter (corn cob) and pre-filled water bottles. Mice received standard diet food (SAFE A03 SP-10, batch U8994G10R00000). All these supplies were sterilized by suppliers.

#### 2.4.1. Collagen-Induced Arthritis Protocol

Arthritis was induced with native bovine collagen type II (CII). Male DBA/1 mice were injected subcutaneously at the base of the tail with 10 mg of CII emulsified in complete Freund’s adjuvant. On day 21, mice have boosted with a subcutaneous (s.c.) injection at the base of the tail with CII in incomplete Freund’s adjuvant. In this model, arthritis usually develops 20–30 days after the first collagen injection [25].

#### 2.4.2. Clinical Score Evaluation

Mice were monitored for evidence of arthritis in their four paws using a blind procedure by a trained operator. For each mouse, the clinical severity of arthritis was scored (0, normal; 1, erythema; 2, swelling; 3, deformity; and 4, ankylosis) in 10 joints or group of joints: three joints of the two hind legs (phalanges, tarsus/metatarsus/metacarpal/carpal, and calcaneus). The maximum score reached for each of the 10 joints was 4, so the maximum score of clinical arthritis reached for a single mouse on a given day was 40. Animals were scored 2 to 3 times per week in the relevant period. The mean arthritic score on each clinical observation day was calculated in each group of treatment. Average severity scores per group and average weight variations per group were analyzed.

#### 2.4.3. Experimental Design

The experimental design for the CIA mice model was the following: treatment groups of 12 animals distributed over all cages (6 animals/cage) to avoid cage effects. Incidence of arthritis was calculated as the percentage of mice of one given group in which the total clinical score was superior to 3. There was always both a naïve and vehicle group.

#### 2.4.4. Mouse Collagen-Induced Arthritis Treatment Scheme

All animals received the same injection volume for treatment, intraperitoneally (i.p.), twice a week (unless otherwise stated). The injected doses for MTX encapsulated in folate-targeted liposomes (FL-MTX) or non-targeted liposomes (L-MTX) are expressed in MTX-equivalent dose. Empty liposomes (folate-targeted–FL, or non-targeted-L) were injected at the same lipid concentration as in FL-MTX and L-MTX. The negative treatment control group was PBS buffer. The positive control group was 35 mg/kg MTX twice a week, with exception of the evaluation of s.c. administration route, which is 7 mg/kg to assess the efficacy at a lower concentration. Varying doses (1, 2, or 4 mg/kg MTX) and frequencies of injection (once or twice a week) of FL-MTX were used and are referred to in the results section and described for each trial. Treatments started on day 14, i.e., one week before the immune boost on day 21, and were continued throughout the testing period, unless stated otherwise.

#### 2.4.5. Nuclear Medicine Imaging

This study was performed by CIPA-Orleans, a public-owned (CNRS) pre-clinical imaging facility specialized in the evaluation of new therapies in vivo, with a specialization in nuclear medicine imaging for the assessment of biodistribution. The study followed radiolabelled liposomes using Single Photon Emitted Computed Tomography (SPECT) imaging in vivo, non-invasively, in the same animals over 4 days after injection. Liposomes were labeled with ^111^Indium (^111^In) and, therefore, the liposomes had to be modified to insert a linker for this purpose. The lipid diethylene-triamine-pentaacetate (DTPA), a chelating agent widely used in nuclear medicine to prepare radiolabelled pharmaceutical agents, was used at 1.5%, ensuring a very effective and specific binding of ^111^In.

A first trial was conducted to ensure that labeling of liposomes incorporating ^111^In was indeed possible with sufficient ^111^In label yield and specificity. Four mice were injected with L-MTX and 4 others with FL-MTX. A SPECT/CT imaging system (Mediso) was used for imaging at the following time points: 30 min, 24 h, 48 h, and 72 h post-injection. At 72 h post-injection, an additional imaging modality was performed with the injection of 550 μCi of Methylene Di Phosphonate (MDP) labeled with ^99m^Technetium (^99m^Tc), allowing dual imaging of liposome (SPECT) and bone metabolism. The separation of ^111^In and ^99m^Tc activity was based on their different decay rate. Mice with established arthritis (day 34 post-immunization) and similar score that had received vehicle were recruited for this study.

Ex vivo CT scan of rear paws was performed to measure paw swelling at the end of the experiment, to correlate paw thickness to liposome accumulation. These higher resolution acquisitions were performed using a Bruker Skyscan 1278 on fixed paws.

#### 2.4.6. In Vivo Biodistribution

Various formulations of MTX were applied to healthy or arthritic DBA 1 mice in the same manner as used in the CIA model (i.p.; s.c. or intravenous, i.v.). At various times after application, blood was sampled from the tail vein. After termination, tissue or cardiac blood was sampled. Blood was separated into plasma using Li-heparin (2 U/mL final concentration) and centrifugation at 4000× *g* for 5 min. For tissue, samples were homogenized in an aqueous buffer containing protease K (1 U/mL, 1 µL buffer per mg tissue) at 37 °C for 2 h. Plasma and tissue homogenates were extracted using acetonitrile (6x volumes with sample weight in mg converted to µL 1:1) containing internal standard (terbuthylazine) (e.g., 10 µL sample + 60 µL acetonitrile). For plasma, the mix was allowed to precipitate on ice for 15 min and then centrifuged (14,000× *g* for 5 min) and the supernatant was placed into HPLC glass vials, suitable for the auto-sampler use. MTX was determined using an HPLC-MS/MS system; Agilent Technologies 1260 Infinity liquid chromatograph equipped with a binary pump and a column oven together with an auto-sampler linked to an AB SCIEX triple-quadrupole mass spectrometry (MS) instrument with an electrospray ionization (ESI) interface. The system is controlled using Analyst Software 1.6.2. from Applied Biosystems Inc. A Raptor Biphenyl (2.7 µm, 50 × 2 mm) chromatography column (Restek) was used for separation.

A standard curve or calibration curve comprising 10 concentrations of MTX (in between 5 nM to 100 µM) was prepared in water. The calibration curve was extracted with 6 volumes of acetonitrile including internal standard. The standard curve was measured twice and was freshly prepared for each analysis. Linear regression analysis of the log peak area versus log theoretical concentration of MTX was used to obtain the apparent sample concentrations which were then corrected for dilution if appropriate. The parent ion for MTX was *m*/*z* H^+^ 455.2 and the fragment measured was *m*/*z* H^+^ 308.3.

### 2.5. Statistical Methods

Pathology development and incidence may provide non-normal distribution of severity scores in the study. A Shapiro test performed on severity scores always shows a significant value confirming this observation. Hence, we used a non-parametric one-way analysis of variance Kruskal-Wallis test at a given time point to test for significant differences in severity score response to treatment between groups. The Dunn post-hoc test without correction was then used to identify differences between all groups. For weight loss, the data could be analyzed using ANOVA followed by a Tukey test to identify differences among groups on a given day. These analyses were performed using R software with the “dunn.test” and multiple comparison libraries for Dunn and Tukey post-hoc tests. A 3rd order polynomial model was used to relate nuclear imaging analysis of the activity in paws to paw swelling. The Wilcoxon rank-sum test was used for in vivo biodistributions studies.

## 3. Results

### 3.1. Study of the Drug-to-Lipid Ratio

**Figure 1:**
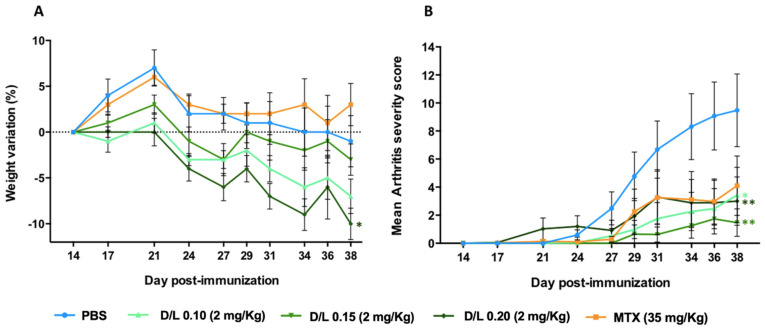
Influence of drug-to-lipid (D/L) ratio on weight loss and therapeutic efficacy of folate-targeted liposomes encapsulating methotrexate (FL-MTX) in collagen-induced arthritis (CIA) mice. (**A**) Average weight variation after treatment. (**B**) Average clinical score as a function of time. All treatments were injected at 2 mg/kg of MTX, twice a week (*n* = 12). Significant differences between liposomes and the vehicle group (PBS) were detected as shown by an * *p* < 0.05 and ** *p* < 0.01.

The development of a liposomal product is quite a complex process as many critical parameters should be investigated during the preparation process. The drug-to-lipid ratio (D/L ratio) is a critical process parameter that represents the capacity of the liposome to accommodate the drug. Thus, this parameter can influence the therapeutic efficacy of the liposomal product, expressing the actual dose of the drug being administrated [26]. Here we investigate the influence of the D/L ratio on adverse effects and therapeutic efficacy of MTX encapsulated in folate-targeted liposomes (FL-MTX) after i.p. administration in the CIA mouse model. We do not evaluate an empty liposome group for this study, which is extensively described in the following experiment. Indeed, unexpectedly some formulations induced loss of weight in some mice due to a lack of feeding. The versatility of the liposomal production method used in this study (a pre-concentrated ethanol injection method) [24] allows the MTX-to-lipid ratio to be greatly varied, with distinct values readily obtained. Three liposomal formulations were prepared with a D/L ratio of 0.10, 0.15, and 0.20, and the therapeutic effect was evaluated at a dose of MTX 2 mg/kg. The liposomal formulations had only limited variation in size and PDI (Supplementary Table S1). All liposome shows a significant difference in total arthritis score reduction (Figure 1B) as compared to the vehicle group (PBS). Weight loss (Figure 1A) are more pronounced for the D/L ratio of 0.10 and 0.20 with a continuous trend along with the experiment, although only the D/L ratio 0.20 liposome shows a significant difference. Therefore, the most favorable D/L ratio is 0.15, showing a negligible weight loss (not significant) and a trend to a better therapeutic effect, compared with the other D/L ratios. A compromise on the initial amount of lipids must be reached so that it is enough to achieve uptake into the target cells and cause the therapeutic effect, but not too high to cause adverse effects. Since the quantity of lipids allowed to be administered to a patient per day is limited, the D/L ratio establishes not only the final drug concentration in the product but also the maximum dose of drug and the number of liposomes that can be administrated per treatment [26]. In this sense, because of the elevated costs, high lipid concentrations may reduce the cost-effectiveness of large-scale manufacturing.

### 3.2. Effective Dose in the Murine CIA Model

**Figure 2:**
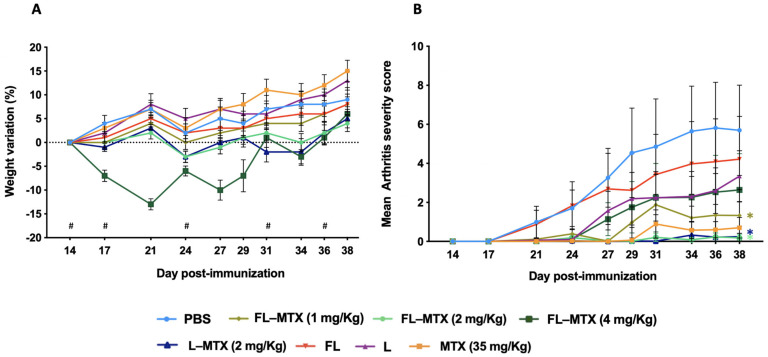
Influence of methotrexate (MTX) dose and folate targeting in collagen-induced arthritis CIA mice. Toxicity and efficacy were assessed after twice-weekly injections of a free soluble form of MTX (MTX) and encapsulated MTX in folate targeted (FL-MTX) or non-targeted (L-MTX) liposomes. Empty targeted or non-targeted liposomes (FL or L) and PBS were used as controls. (**A**) Average weight variation after injection of treatments. Each graph shows the average weight variation after day 14 (time of the first injection of treatments) among groups of mice treated (*n* = 12). In the 4 mg/kg dose, the frequency was reduced to once weekly (# assigned in the graphs). (**B**) Mean arthritis severity (*n* = 12). Significant differences between liposomes and the vehicle group (PBS) were detected as shown by an * *p* < 0.05.

To evaluate the most effective dose to prevent arthritis development, CIA mice were injected with several liposomal formulations at an MTX dose of 1, 2, or 4 mg/kg, twice a week. Results showed that the 4 mg/kg dose of FL-MTX rapidly induced weight loss in the animals (Figure 2A), leading us to switch, during the experiment (for ethical and practical reasons), from twice to once-weekly injection. This severe weight loss is most probably due to reduced food consumption. However, the dose of 4 mg/kg injected once a week was not as effective as 2 mg/kg given twice a week (Figure 2B; no significant difference for 4 mg/kg against vehicle PBS group). A 1 mg/kg dose injected twice a week had mild efficacy, also significant against the vehicle PBS group. Recently, Chen et al. used a liposomal formulation to deliver MTX for the treatment of arthritis in C57BL/6 CIA mice [27]. A similar decrease of the arthritic score was seen after i.v. injection of 1 mg/kg of MTX once every two days for a total of five injections. Liposomes encapsulating MTX were also used in Lewis CIA rats via daily i.v. injection of 2.5 mg/kg MTX for 4 days [28].

Non-targeted liposomes (L-MTX) at a dose of 2 mg/kg given twice a week were similar to FL-MTX in apparent efficacy. While folate targeting in FL-MTX has a better impact on the reduction of the clinical signs in terms of comparison of the crude mean, this difference is not statistically significant due to the non-normal distribution of clinical scores.

A clinical follow-up was performed for a few days after the experiment in which the mice were untreated. This relapse trial showed that mice treated with FL-MTX had increased arthritis scores after the treatment was stopped (Supplementary Figure S1). These data suggest that the disease is responding to therapy, however, a population of pathological autoreactive T-cells are present (due to immunization) and that these are capable of inducing arthritis in the absence of treatment. In so far as MTX acts by preventing T-cell proliferation, once levels of MTX decline after cessation of treatment, it will be possible for auto-reactive T-cells to resume proliferation.

In the normal time-course of CIA development, the initial reaction to exogenous CII results in a stimulation of reactive T-cells and a corresponding T-regulator response. Boost and or stimulation of myeloid cells causes regulatory T cells (Tregs) to be less effective and pro-inflammatory cytokines to predominate leading to inflammation in tissues where both antibodies aggregate, and antigen is located. By day 14, the production of autoreactive T-cells has already started. Thus, this initial process of antigen presentation and clonal selection and proliferation cannot be modulated by the therapy after day 14. Therapy can, however, influence, homing and activation of these cells, and the proliferation of reactive T-cells.

The rapid onset of arthritis a few days after the end of treatment, confirms that the induction of arthritis was effective but that the expansion of the auto-reactive cells was controlled by treatment with FL-MTX. This form of analysis is less relevant for Vehicle animals in that proliferation has already occurred and those with high scores cannot further increase scores within the acceptable ethical parameters, while those with low scores were intrinsically less susceptible to CIA (pre-selection error). There is some effect of i.p. injections on overall immune stimulus (the peritoneum is a sensitive compartment with many myeloid cells and injections lead to IL-6 secretion), thus discontinuation of saline is unlikely to lead to additional immune stimulus in animals with low disease states.

### 3.3. Targeting of Inflamed Paws

**Figure 3:**
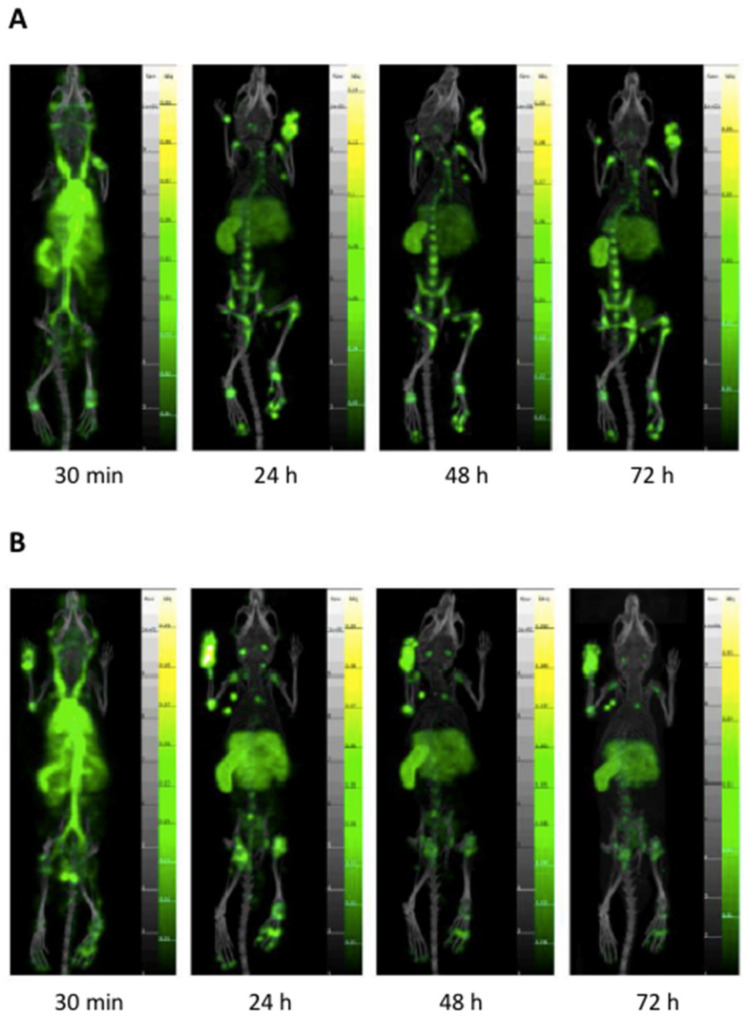
Pharmacokinetics of ^111^In-labelled liposomes encapsulating methotrexate (MTX) assessed by nuclear imaging Single Photon Emitted Computed Tomography (SPECT-CT). (**A**) Non-targeted liposomes (L-MTX). (**B**) Folate-targeted liposomes (FL-MTX). Please note at 30 min the early targeting of inflamed joints and the strong signal from vessels. Targeting in inflamed joints remains very stable over time in inflamed joints.

We have shown that FL strongly accumulates in the joints of the arthritic mice, using whole-body mouse fluorescence imaging [23]. Since in vivo fluorescence is not quantitative, we assessed liposome pharmacokinetics by nuclear imaging (SPECT-CT) through the inclusion of 1.5% of DTPA allowing radiolabelling with ^111^In [29]. After initial validation of the imaging protocol, showing no uptake of ^111^In-labelled liposomes in healthy joints (data not shown), the pharmacokinetics in CIA mice was followed for 72 h. FL-MTX reached inflamed joints within 30 min post-injection (Figure 3) and were still present 72 h after injection. However, no significant difference is observed in the concentration of liposomes in paws with or without folate targeting. In contrast, the FL-MTX appears to enter the spleen and liver more rapidly and label both organs differently. The L-MTX signal increases in the spleen more slowly. FL-MTX also appears to concentrate to a greater extent in proximal lymph nodes is affected paws.

Liposome accumulation can be linked to various mechanisms. Neoangiogenesis is well known in inflammatory pannus in both inpatients and CIA mice, leading to a higher vascular density [30,31], which provides better distribution of the liposomes in the arthritic joints. Furthermore, vessels in arthritic joints are more permeable allowing higher rates of extravasation of liposomes and accumulation in the joint [32,33]. Finally, numerous immune cells present in the inflamed joints can phagocytose the liposomes, leading to an enhanced uptake in the joint [34,35,36].

**Figure 4:**
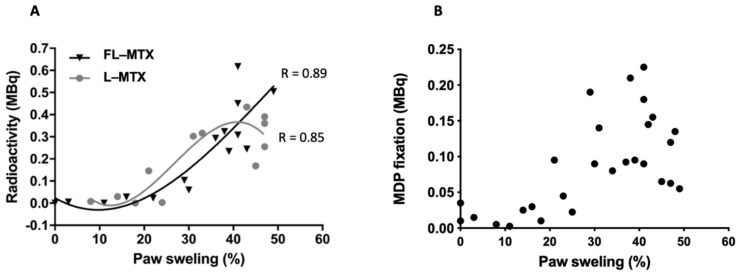
Specificity of liposomes encapsulating methotrexate (MTX) to arthritic joints. (**A**) Liposome accumulation is a function of paw swelling. The accumulation of liposomes directly correlates with the percentage of swelling. (**B**) Methylene Di Phosphonate (MDP) activity as a function of paw swelling, strongly indicating bone erosion activity.

The association between the degree of paw swelling and the accumulation of liposomes was assessed. The percentage swelling of the arthritic paws was calculated using as reference the healthy front and rear paws. Then, liposome accumulation was plotted against paw swelling for both FL-MTX and L-MTX (Figure 4A). A strong accumulation of liposomes can be measured as the paws swell, which confirms the affinity of liposomes to arthritic tissue as non-arthritic paws does not retain the liposomes. Once again, it is not possible to observe the influence of folate targeting on the accumulation of liposomes in the paws.

To evaluate the bone remodeling activity, ^99m^Tc MDP, a radiotracer used in nuclear medicine for bone scans [37] was injected in these animals (Supplementary Figure S2). Bone remodeling activity partly matched with the paw swelling index, reflecting the occurrence of mechanisms related to bone erosion (Figure 4B).

### 3.4. MTX Distribution in Non-Targeted Tissues

**Figure 5:**
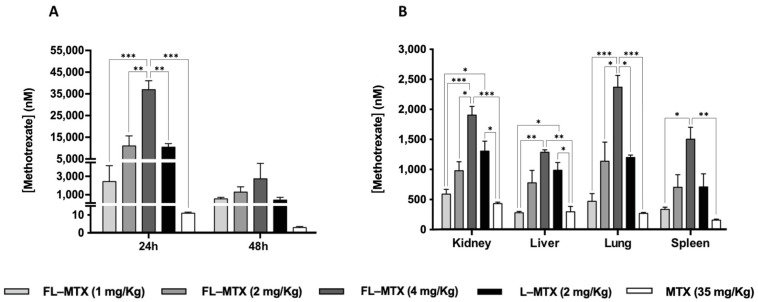
Distribution of FL-MTX in a dose-dependent manner compared to non-targeted liposomal MTX (L-MTX) and free MTX (MTX). (**A**) Serum MTX concentration at 24 h and 48 h post i.p. injection. (**B**) MTX concentration in non-target tissues 24 h post i.p. injection. Significant differences between all groups (liposomes and MTX) were detected as shown by an * *p* < 0.05, ** *p* < 0.01 and *** *p* < 0.001.

In vivo biodistribution of different liposomal formulations after i.p. administration was evaluated by quantifying MTX in serum and several non-target organs. In naïve mice, biodistribution reveals that the absence of folate targeting leads to increased MTX delivery at 24 h in bile, kidneys, spleen, and possibly in the gut (Supplementary Figure S3). In CIA mice, encapsulated MTX remains in serum from 24 h to 48 h. The extended presence of MTX in serum is most probably indicative of intact liposomes circulating rather than free MTX which is normally rapidly cleared [38]. Tissue concentration of MTX is also higher when the drug is encapsulated in liposomes as opposed to the free soluble form of MTX, even though the dose used is much lower (2 mg/kg in liposomes as opposed to 35 mg/kg in free MTX). There is a close dose-dependency between the number of liposomes injected and the measured MTX concentration in serum (Figure 5A, linear relationship for FL-MTX at 1, 2, and 4 mg/kg, at 24 h, correlation coefficient R = 0.92) and various non-target organs (liver, spleen, kidneys, lungs) (Figure 5B, correlation coefficients R = 0.87, 0.86, 0.93 and 0.89, respectively). The dose delivered has an impact on liposomal treatment efficacy in CIA mice as well as on possible adverse effects.

Indeed, one other aspect of liposome targeting concerns non-target organs. At 24 h post-injection MTX there is a trend for a higher concentration in the liver or kidneys for L-MTX (Figure 5B) as opposed to L-MTX at the same dose, whereas no differences could be observed in the serum between L-MTX and FL-MTX (Figure 5A). These results could indicate that folate-targeting of liposomes induces pharmacokinetic changes by reducing the partition to hepatic and renal tissues which are responsible for nanoparticle elimination [39,40,41].

### 3.5. Subcutaneous Injection Route

**Figure 6:**
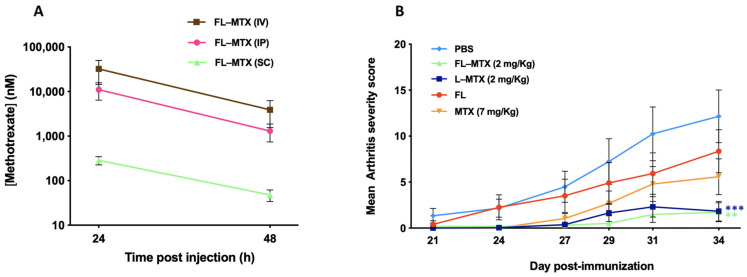
Influence of s.c. route of administration in collagen-induced arthritis (CIA) mice. (**A**) Methotrexate (MTX) serum concentration after 2 mg/kg administration of FL-MTX by in i.p., s.c. or i.v. injection. (**B**) Efficacy of 2 mg/kg of liposomal MTX (FL-MTX and L-MTX) given by s.c. injection. Free MTX was administered at a 7 mg/kg dose *s.c.* and PBS was also injected s.c. (*n* = 12). Significant differences between liposomes and the vehicle group (PBS) were detected as shown by ** *p* < 0.01 and *** *p* < 0.001.

Although the parenteral administration in animal models is commonly i.p. (frequently the i.v. route is not feasible for technical reasons) [42], this route is less suitable for long-term human therapy. For liposomes, in particular, the s.c. route of administration is preferable, not only because it is a simpler route for patient’s self-administration, but also because it might serve as a depot for the sustained drug release in vivo [43]. With the behavior of FL-MTX well established in the CIA model when administered by i.p. injection, we wanted to assess the efficacy and toxicity of our liposomal MTX after *s.c.* administration. To determine the target dose to inject in animals, the concentration of MTX in serum was determined after 24 h and 48 h post i.p., i.v. and s.c. injection. The results show that, compared with the i.p. route, MTX serum concentration was lower via the s.c. route (1.5 log_10_ lower, Figure 6A), which led us initially to increase the injected dose. However, severe weight losses in CIA mice were registered after the s.c. injection of FL-MTX at 8 and 16 mg/kg (data not shown). By contrast, soluble MTX showed no toxicity effect even at a very high concentration (16 mg/kg). Not surprisingly, MTX serum concentration is not a good indicator of MTX bioavailability in mice injected with the drug encapsulated into liposomes. Indeed, Allen and colleagues reported that liposomes levels were significantly higher in the draining lymph nodes after s.c. administration and concentrations in other tissues were proportionately reduced relative to the i.v. and i.p. injections [43]. Furthermore, another study found that the blood concentration of a pegylated liposomal formulation 24 h after the s.c. injection is much lower (approx. 30 times) than after i.v. injection [44].

Administering the same 2 mg/kg dose by s.c. injection proved to be as effective as the i.p. injection in CIA mice. FL-MTX and L-MTX showed similar efficacy and performed better than free MTX (7 mg/kg) which was given in a 3.5 times higher dose (Figure 6B).

## 4. Conclusions

Folate-targeted liposomal formulation encapsulating MTX (FL-MTX) provides new pharmacological properties to MTX. In particular, a far lower dose of MTX is required for a given reduction in arthritic score. At the same time, the formulation can impact animal food intake and this effect appears to depend on the D/L ratio (optimal was 0.15) but the reason for this effect remains unclear. The optimal dose was 2 mg/kg twice weekly; 1 mg/kg twice weekly was not effective while 4 mg/kg twice weekly was not tolerated.

Imaging revealed that the liposomes accumulate in inflamed joints in proportion to the paw swelling and bone remodeling activity. The pharmacokinetics of MTX is modified by its encapsulation in the liposomes, increasing the circulation time, however, this was not dependent on folate targeting. Finally, to simulate the preferred clinical route, we compared *s.c.* injection to *i.p.* The same dose was equally effective by both the s.c. and i.p. routes.

These data suggest that folate-targeted liposomes are a stable form of liposome for MTX delivery. The apparent potency of MTX increases as a liposome with 2 mg/kg equivalent in efficacy to 35 mg/kg of the free substance. While this approach will potentially allow a reduction of the dose used clinically, there remains the issue that new effects are associated with this form, notably loss of food intake. While such effects are likely to be specific to mice, it remains important to carefully escalate from very low doses in such cases.

Animal models also have the inherent issue that the stage of the disease varies over a short time scale and that the phase of initiation of therapy impacts results. In patients, MTX is used mainly to maintain remission and so there is a degree of systemic as well as local action. It remains to be seen to what extent the advantages of the liposomal form relate to targets in major immune organs like to spleen or exposure to inflammatory lesions. Should the formulation be more effective and tolerable, it represents at least one means to replace more expensive therapy forms.

## Supplementary Materials

The following supporting information can be downloaded at: https://www.mdpi.com/article/10.3390/biomedicines10020229/s1, Figure S1: Relapse of collagen-induced arthritis (CIA) arthritis upon the arrest of treatment, Figure S2: Example of bone metabolism imaging and liposome distribution in arthritic mouse, Figure S3: Biodistribution of FL-MTX compared to non-targeted liposomal MTX (L-MTX), in naïve mice. Table S1: Characterization of liposomes used in this work.
