# Supplementary material for: Folate-Targeted Liposomal Formulations Improve Effects of Methotrexate in Murine Collagen-Induced Arthritis
Source: Biomedicines. 2022 Jan 21;10(2):229. doi: 10.3390/biomedicines10020229 (PMC8869739; doi:10.3390/biomedicines10020229)
Supplement: Supplementary file 1 [file biomedicines-10-00229-s001.zip › biomedicines-1530241-supplementary.pdf]

biomedicines

Table S1. Characterization of liposomes used in this work.

Liposomal Formulation	Labelling	Drug-To-Lipid Ratio	Z-Average (d.nm)	Polydispersity Index
FL-MTX	-	~0.10	130.6 ± 1.097	0.097 ± 0.041
	-	~0.15	138.0 ± 0.351	0.092 ± 0.022
	-	~0.20	121.2 ± 0.473	0.086 ± 0.009
L-MTX	-	~0.15	129.7 ± 0.115	0.075 ± 0.010
FL	-	-	117.3 ± 1.917	0.162 ± 0.004
L	-	-	143.2 ± 0.757	0.057 ± 0.026
FL-MTX	DTPA-^{111}In	~0.15	100.1 ± 1.294	0.148 ± 0.005
L-MTX	DTPA-^{111}In	~0.15	99.71 ± 2.157	0.134± 0.016

FL-MTX: folate-targeted liposomes encapsulating methotrexate; L-MTX: non-targeted liposomes encapsulating methotrexate; FL: folate-targeted liposomes; L: non-targeted liposomes; DTPA: diethylene-triamine-pentaacetate.

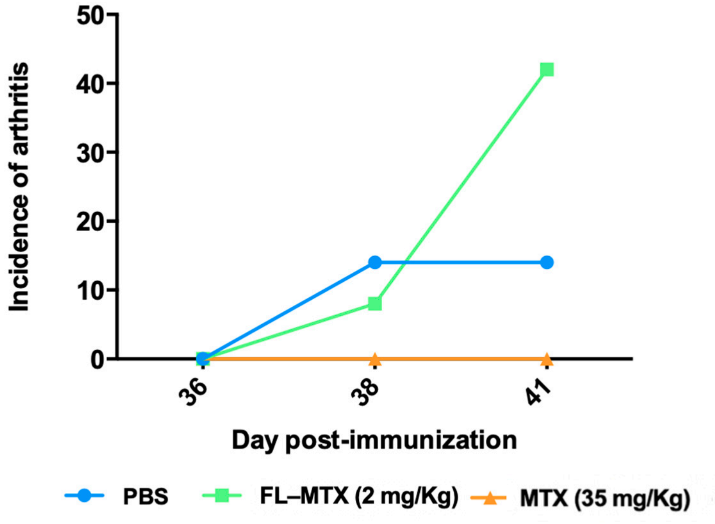

Figure S1. Relapse of collagen-induced arthritis (CIA) arthritis upon arrest of treatment. Incidence of pathology is expressed in percentage of CIA mice of each group that had no clinical signs of arthritis at day 34 (last treatment injection).

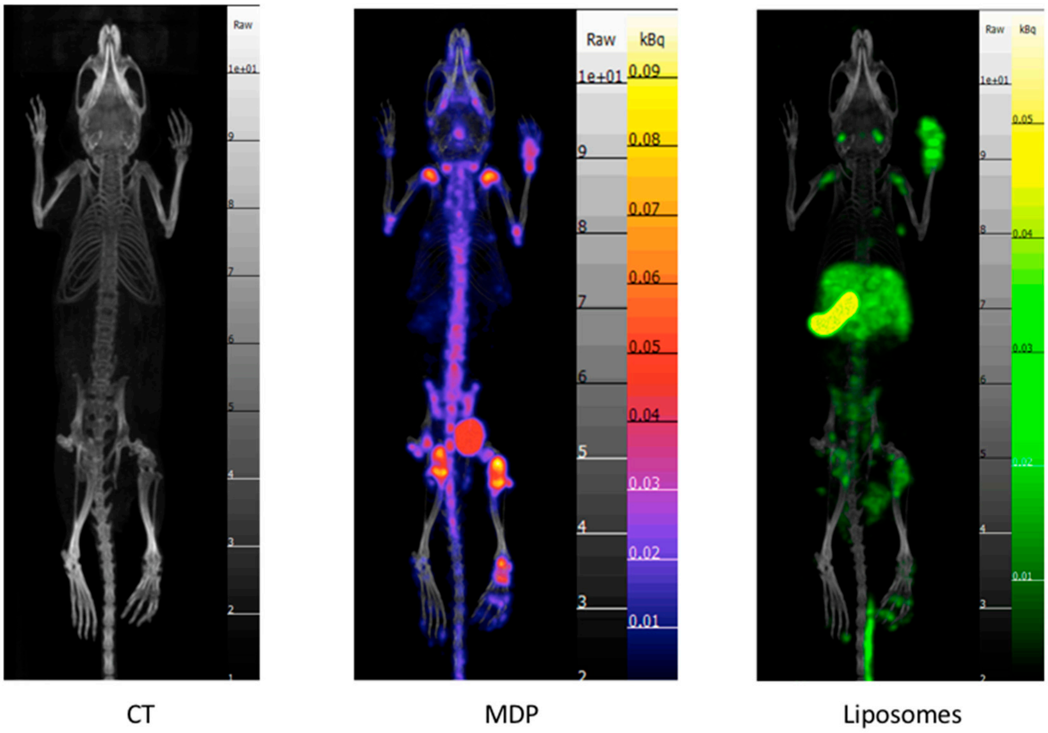

Figure S2. Example of bone metabolism imaging and liposome distribution in arthritic mouse. The left image shows the Computed Tomography (CT) scanner of the animal, the middle one, the combination of Scanner and Methylene Di Phosphonate (MDP)-^{99m}Tc activity and the right one, combination of CT-scanner and FL-MTX-DTPA-^{111}In, 72 h post injection. MDP reveals bone remodeling activity that correlates with accumulated FL-MTX liposome in joints. Please note that non arthritic joints show no or very limited activity in both MDP and FL-MTX distribution.

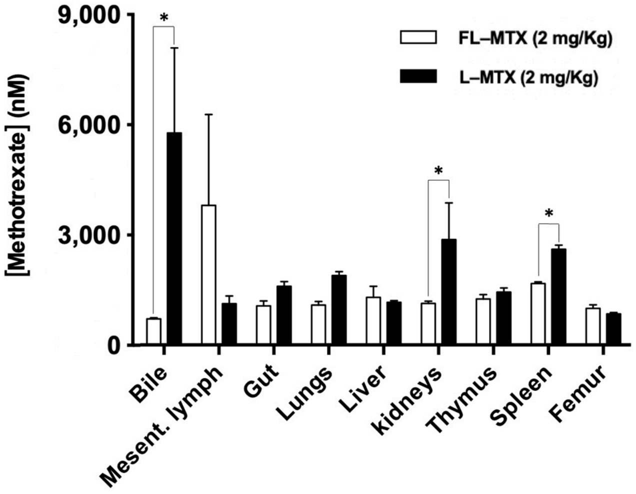

Figure S3. Biodistribution of FL-MTX compared to non-targeted liposomal MTX (L-MTX), in naïve mice. MTX concentration in non-target tissues 24 h post i.p. injection ($n = 5$). * Indicates significant differences ($p < 0.05$) in Wilcoxon rank sum test.
